# Prediction of Metastasis in Small Pulmonary Oligonodules Detected in Breast Cancer Patients at Baseline CT

**DOI:** 10.1002/kjm2.70014

**Published:** 2025-03-27

**Authors:** Huei‐Yi Tsai, Min‐Fang Chao, Tzu‐Hsueh Tsai, Shen‐Liang Shih, Fang‐Ming Chen, Ming‐Feng Hou, Ming‐Yii Huang, Jui‐Sheng Hsu

**Affiliations:** ^1^ Department of Medical Imaging Kaohsiung Medical University Hospital, Kaohsiung Medical University Kaohsiung Taiwan; ^2^ Center for Big Data Research Kaohsiung Medical University Kaohsiung Taiwan; ^3^ Graduate Institute of Clinical Medicine College of Medicine, Kaohsiung Medical University Kaohsiung Taiwan; ^4^ Division of Breast Oncology and Surgery Department of Surgery, Kaohsiung Medical University Hospital, Kaohsiung Medical University Kaohsiung Taiwan; ^5^ Department of Surgery Kaohsiung Municipal Ta‐Tung Hospital, Kaohsiung Medical University Kaohsiung Taiwan; ^6^ Department of Surgery Faculty and College of Medicine, Kaohsiung Medical University Kaohsiung Taiwan; ^7^ Department of Biomedical Science and Environmental Biology College of Life Science, Kaohsiung Medical University Kaohsiung Taiwan; ^8^ Department of Radiation Oncology Kaohsiung Medical University Hospital, Kaohsiung Medical University Kaohsiung Taiwan; ^9^ Department of Radiation Oncology School of Medicine, College of Medicine, Kaohsiung Medical University Kaohsiung Taiwan; ^10^ Department of Radiology School of Medicine, College of Medicine, Kaohsiung Medical University Kaohsiung Taiwan

**Keywords:** breast neoplasms, computed tomography, lung neoplasms, multiple pulmonary nodules, solitary pulmonary nodule

## Abstract

It is challenging for radiologists to diagnose pulmonary metastases when they encounter only a few (*n* ≤ 5) small pulmonary nodules (< 10 mm) on staging CT in breast cancer patients. We conducted this study to assess clinical and imaging features related to metastasis for better risk stratification. Retrospective analysis of 249 pulmonary nodules present at the baseline CTs of 194 patients diagnosed with breast cancer between 2014 and 2021 was performed. The evaluated features included nodular characteristics, perifissural nodules, associated imaging findings, clinical stage, and breast cancer subtype. Nodules with interval change were determined to be metastases. A large proportion of the patients had single nodule (78.9%) presence, and most of the nodules were less than 6 mm (86.3%). Among the 249 nodules, 63 (25.3%) nodules were in metastases. The independent predictors were nodule ≥ 6 mm, mediastinal/hilar lymphadenopathy, clinical Stages III and IV, and triple‐negative breast cancer subtype. Nodules (≥ 6 mm) were assessed as weak evidence to rule in metastasis, and the results were as follows: positive likelihood ratio (+LR), 3.74; sensitivity, 30.2%; and specificity, 91.9%. With weak evidence of small pulmonary nodules (≧ 6 mm) to rule in metastases, it may be appropriate to follow the recommendations of growing nodule management. By contrast, the nodular shape, margin, location, perifissural nodules, and pleural tag did not show an association with metastasis.

## 1 Introduction

According to the World Health Organization, breast cancer is the most prevalent cancer globally [1], with the incidence of breast cancer ranking first in 85% of countries. In 2020, there were 2.3 million newly diagnosed breast cancer cases and 68.5 thousand breast cancer deaths. The 5‐year disease‐specific survival rate in stage IV breast cancer is 35.5%, which is significantly different from that in Stages I–III (98.5%–70.8%) [2]. Both work‐up and treatment strategies for breast cancer with distant metastases and non‐metastases are different [3]; therefore, accurate detection of distant metastases is important for treatment planning.

The most common sites for metastatic breast cancer are bones, lungs, liver, and brain [4]. Computed tomography (CT) is the most important imaging modality for the evaluation of pulmonary metastasis, with typical findings as multiple peripherally located round nodules and lower lobe predominance [5]. Yang et al. [6] studied the predictors of metastasis in small pulmonary nodules (≤ 10 mm) detected in patients with primary extrapulmonary malignancy and found an increasing odds ratio of metastasis along with an increase in nodular number. Hammer et al. [7] conducted a study to find the predictive factors for malignancy, including metastases of breast cancer and other malignancies, of the nodules on baseline CT of breast cancer patients, where nodular number was also reported to be an independent predictor of metastasis; additionally, the malignancy rate of the pulmonary nodules presented on baseline CT was low, ~13%. With a low malignancy rate, it is more challenging for radiologists to diagnose metastasis when facing only a few small nodules in the lung—a situation encountered frequently in daily practice.

The perifissural nodule (PFN) was coined to represent the CT presentation of intrapulmonary lymph nodes and is typically considered benign in lung cancer screening [8]. However, their significance and optimal management in oncological patients remain unclear. To our knowledge, no published study has evaluated whether PFNs can be confidently classified as benign lesions in breast cancer patients. In patients with a few pulmonary nodules and a breast cancer, it remains unclear whether such patients should be considered for further adjuvant therapy and/or intense follow‐up by imaging. Therefore, this study aimed to review chest CT scans of breast cancer patients with small oligonodules (< 10 mm, *n* ≤ 5) and to identify clinical and imaging factors associated with pulmonary metastasis.

## 2 Materials and Methods

### 2.1 Study Subjects

**FIGURE 1:**
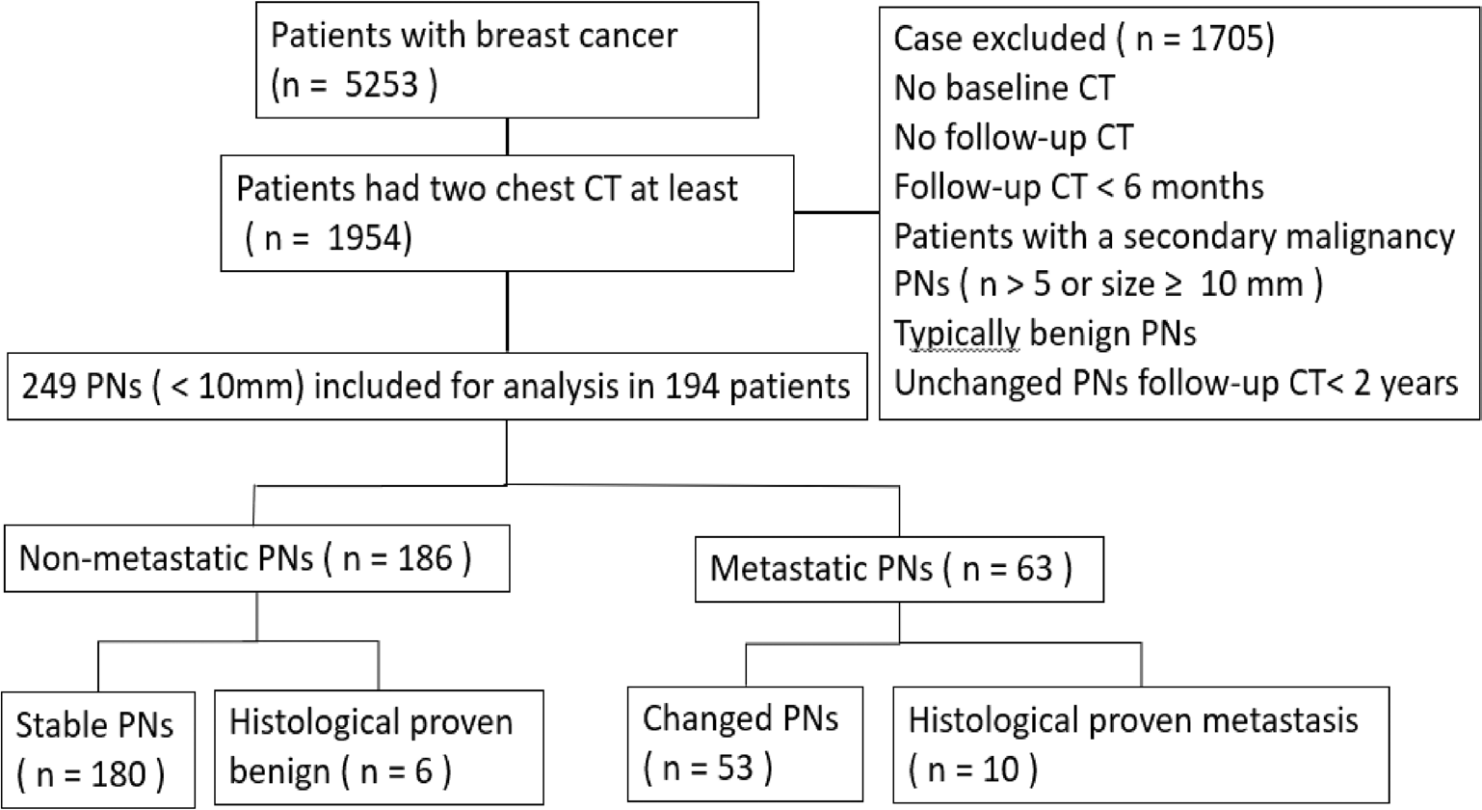
The flow chart shows the strategy for recruitment of the patients. PNs are pulmonary nodules.

This retrospective study was approved by the Institutional Review Board of our hospital. The requirement for written informed consent was waived. From January 2014 through December 2021, 5253 invasive breast cancer patients were registered in the cancer registration database of our institution, but only patients with baseline chest CT and at least one follow‐up CT were enrolled (*n* = 1954), regardless of whether a contrast agent was administered or not. Baseline CT was defined as the CT scan performed within 3 months before or after breast cancer diagnosis. The upper limit of the time interval between 2 CT scans for metastasis was 2 years. Follow‐up CT should be performed at least 6 months after baseline CT. One radiologist (a 25‐year experience in chest imaging) reviewed the baseline CTs and electronic medical records, selecting study subjects based on the inclusion and exclusion criteria. The inclusion criteria were at least one but fewer than six solid pulmonary nodule(s) being less than 1 cm in size on CT images. The exclusion criteria were as follows: (1) no available complete pathologic information of the breast cancer; (2) the patient had a second malignancy; (3) nodules had typically benign features such as intra‐nodular fat or benign‐type calcification; (4) ground‐glass opacity because of its extremely low possibility of metastasis from breast cancer [9]; (5) and nodules with dependent posterior subpleural attachment because we could not differentiate nodule atelectasis from true nodule without prone CT scanning [10]. Pulmonary nodules detected at baseline CT were evaluated and followed until the last available follow‐up CT, while each nodule was analyzed on baseline CT, but nodules that developed over time were not analyzed (Figure 1).

### 2.2 Image Acquisition

Several multidetector‐row CT scanners, including Somatom Definition Flash/Sensation 16 (Siemens Medical Systems, Forscheim, Germany), Brilliance 64 (Philips Medical Systems, Haifa, Israel), and Toshiba Aquilion One TSX‐301 (Nasu, Japan) were used for scanning. Most of the baseline CTs were performed for breast cancer staging, but some patients underwent study for the evaluation of other possible pulmonary or cardiovascular diseases. At our institution, chest CT is reconstructed with 1.5–2.5 mm slice thickness for axial images and 5 mm for coronal and sagittal planes. A small number of CTs were acquired from other institutions because the patients were first diagnosed there. These images were reconstructed with 2.5–5 mm slice thickness.

### 2.3 Pulmonary Nodules Evaluation

Nodules on the images with less than 3 mm slice thickness were evaluated except for a small number of images performed at other institutions (only 5 mm images were available). The nodules should be clearly depicted and measurable on at least two different reformatted planes. All the images were reviewed on our picture archiving and communication system (EBM, Taipei, Taiwan), which allowed us to compare the same nodules on baseline and follow‐up images side by side. The pulmonary nodules were evaluated on lung window (level − 600 HU; width 1500 HU) while the mediastinum was evaluated on soft tissue window (level 40 HU; width 400 HU). Two experienced radiologists with 25 and 10 years of experience in thoracic radiology, respectively, reviewed the images and recorded the size and characteristics of the nodules with consensus. Both radiologists were aware that the patients had breast cancer but were blinded to the clinical staging or details in pathology reports.

### 2.4 Nodule Characteristics

Five nodular features were recorded: location, size, shape, margin, and pleural/fissure attachment. The location of the pulmonary nodules was classified as being on the left or right, being above or below the carina, or being in the upper, middle (lingual) or lower lobes. The size was measured on the axial image showing the maximum area of the nodule. The average of the maximum length and maximum width perpendicular to the length was used. Additionally, we divided size into < 6 mm and ≥ 6 mm with rounding the average nodule diameter to the nearest whole millimeter. Consequently, we rounded a 5.5 mm nodule up to 6 mm, while a 5.4 mm nodule was rounded down to 5 mm.

The shape was classified into round (width divided by the length was lesser than 0.66), oval/lentiform (nodule border with convex toward the lung and width divided by the length was greater than 0.66), triangular, and polygonal (nodule borders with the lung were straight or concave). A nodule might reveal more than one shape on different reconstructed planes. In this situation, the shape was prioritized in the following order: polygonal, triangular, oval/lentiform, and round. For example, we categorized the shape as oval/lentiform when one nodule showed oval/lentiform and was round on different reformatted images. The margin was considered either smooth or non‐smooth (irregular, abrupt bulging, or spiculated). Four categories of pleural/fissure attachment were defined: (a) no attachment; (b) pleural attachment (abutting on the pleurae of chest wall or mediastinum without on the fissures); (c) fissure attachment (abutting on fissures with/without simultaneously on the pleura); and (d) attachment to vessels (artery or vein).

We also classified the nodules as perifissural nodule (PFN) or otherwise. PFN was defined as a pulmonary fissure‐attached solid nodule with an oval/lentiform or triangular/polygonal shape in at least two orthogonal planes and a maximum diameter of less than 10 mm [4, 8].

### 2.5 Associated Imaging Findings and Clinical Information

Several associated imaging findings were recorded, including the presence of pleural effusion, mediastinal/hilar lymphadenopathy (a lymph node with short axis longer than 10 mm or conglomerated lymph nodes), and pulmonary nodules with pleural tags to fissure, chest wall, or mediastinal pleurae. Clinical information, including age, AJCC 8th cancer staging, and pathological information such as tumor grade and cancer subtype, was obtained from the institutional cancer registration database. The cancer subtype was classified based on the immunohistochemistry test into four categories: (1) Hormone receptor (HR)‐positive and human epidermal growth factor receptor 2 (HER2)‐negative; (2) HR‐positive and HER2‐positive; (3) HR‐negative and HER2‐positive; or (4) Triple‐negative breast cancer (TNBC).

### 2.6 Determination of Metastases

**FIGURE 2:**
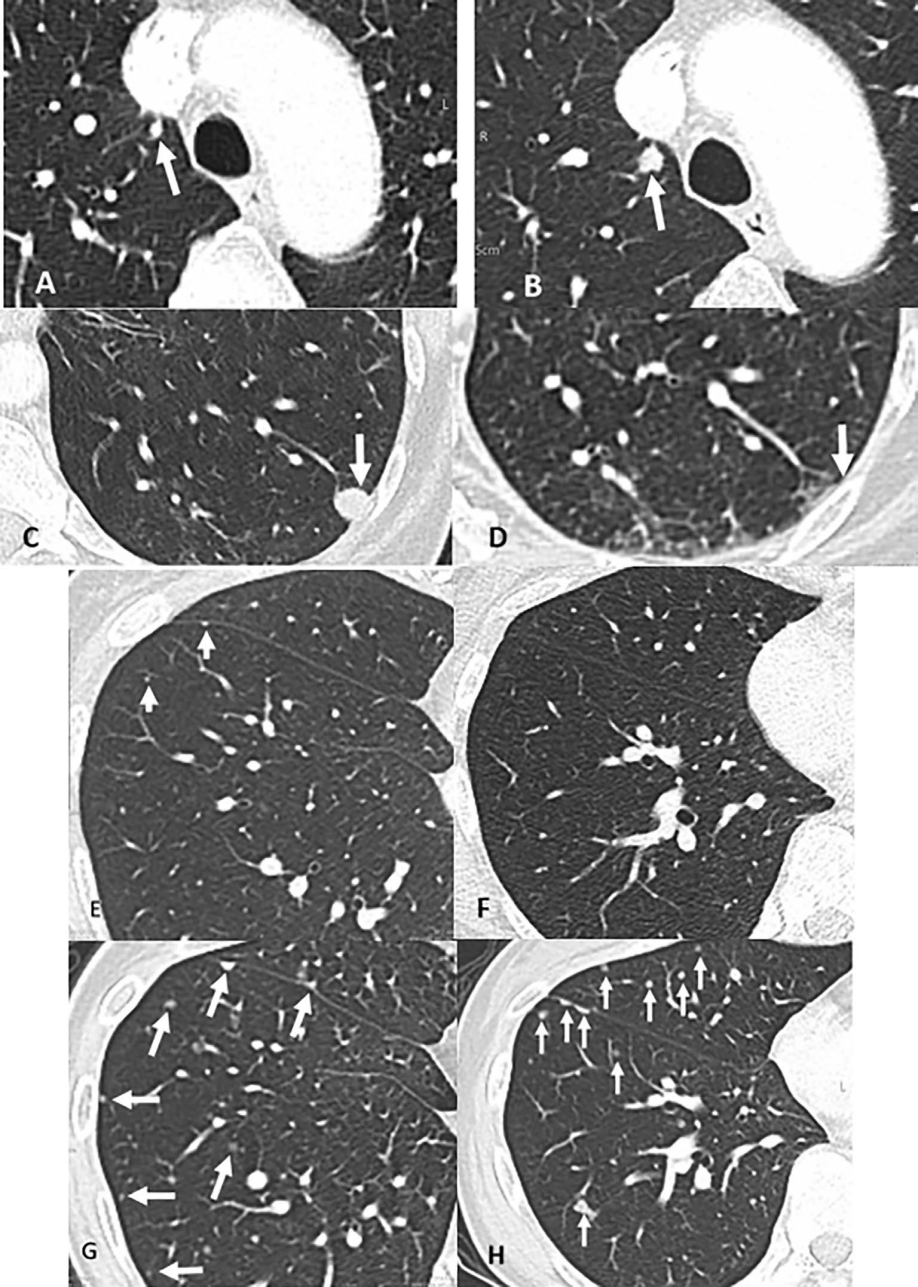
Illustrations of metastatic PNs. (A, B) A round nodule in the right upper lobe (white arrow), measured 3 mm, following up by 167 days after the initial CT, the nodule increased gradually. (C, D) A round nodule in the left lower lobe (white arrow), measured 9 mm, following up by 136 days after the initial CT, the nodule disappeared. (E–H) A perifissural nodule and a small round nodule(white arrow) (E, F), measured 1 mm, following up by 180 days after initial CT, increased the size of and number of small metastatic nodules and perifissural nodules (G, H).

With underlying malignancy, pulmonary nodules demonstrating radiological evidence of interval changes on serial images are more likely to be metastasized [6]. Interval size change, including increase and decrease in size or even disappearance, was defined as metastasis because the size may increase as the disease progresses or decrease owing to the effect of systemic therapy. We classified a pulmonary nodule as in metastasis if it disappeared or showed change in size ≥ 2 mm (persistent increase or shrinkage or increase followed by decrease or decrease followed by increase in size) after the initial CT examination (Figure 2). A stable nodule (size change less than 2 mm) at least 2 years CT follow‐up, by contrast, was considered to be a non‐metastatic nodule. In addition, a small number of nodules were classified according to the pathological reports after surgery.

### 2.7 Statistics

Pearson Chi‐square test and independent *t*‐test were used to compare the features between metastatic and non‐metastatic nodules, while univariate and multivariate logistic regression analyses were used to assess the relationships among various variables and pulmonary metastases. Odds ratio (OR) was obtained with a 95% confidence interval (CI), with a *p*‐value < 0.05 defined as statistically significant. Additionally, diagnostic accuracy, sensitivity, specificity, positive predictive value (PPV), negative predictive value (NPV), and positive likelihood ratio (+LR) were calculated with nodule (≥ 6 mm) for prediction of metastasis. We rated +LRs as follows: greater than 10, strong evidence to rule in metastasis; 5–10, moderate evidence to rule in metastasis; 2–5, weak evidence to rule in metastasis; 0.5–2.0, no significant change in the likelihood of metastasis; 0.2–0.5, weak evidence to rule out metastasis; 0.1–0.2, moderate evidence to rule out metastasis; and less than 0.1, strong evidence to rule out metastasis.

## 3 Results

### 3.1 Patient Characteristics

One hundred and ninety‐four females (mean age 53 ± 11 years, range 29–85) with 249 pulmonary nodules were enrolled in this study. A large proportion of the patients had a single nodule only (78.9%). The incidence of metastases increased along with the increase in clinical stage: 8.6% (5/58) in Stage I, 16.9% (12/71) in Stage II, 33.3% (7/21) in Stage III, and 50.0% (22/44) in Stage IV. The demographic data, clinical TNM stage, and histological details of the primary breast cancer have been summarized in Table 1. Forty‐four patients had Stage IV disease at diagnosis, of whom 22 patients had non‐visceral metastases (bone, skin, and lymph node), 12 patients had visceral metastases, and 10 patients had visceral and non‐visceral metastases. In the clinical and pathological variables, only the clinical stage showed a statistically significant difference between metastatic and non‐metastatic groups (*p* < 0.001).

**TABLE 1:** Demographic data, clinical TNM staging, and histological details of the primary breast cancer.

	Patients with non‐metastatic nodules (*N* = 148, 76.3%)	Patients with metastatic nodules (*N* = 46, 23.7%)	Total (*N* = 194)	*p*
**Age (mean ± SD)**	53.4 ± 10.8	53.2 ± 10.1	53.4 ± 10.6	0.888
**Number of nodules**				0.377
1	120 (81.1)	33 (71.7)	153 (78.9)	
2	18 (12.2)	9 (19.6)	27 (13.9)	
3	10 (6.8)	4 (8.7)	14 (7.2)	
**Clinical stage**				< 0.001
1	53 (35.8)	5 (10.9)	58 (29.9)	
2	59 (39.9)	12 (26.1)	71 (36.6)	
3	14 (9.5)	7 (15.2)	21 (10.8)	
4	22 (14.9)	22 (47.8)	44 (22.7)	
**Tumor grade**				0.089
1	7 (4.7)	1 (2.2)	8 (4.1)	
2	93 (62.8)	22 (47.8)	115 (59.3)	
3	48 (32.4)	23 (50.0)	71 (36.6)	
**Breast cancer subtype**				0.163
Hormone receptor (+), HER2 (−)	88 (59.5)	21 (45.7)	107 (56.2)	
Hormone receptor (+), HER2 (+)	34 (23.0)	10 (21.7)	44 (22.7)	
Hormone receptor (−), HER2 (+)	17 (11.5)	9 (19.6)	26 (13.4)	
Triple negative	9 (6.1)	6 (13.0)	15 (7.7)	

Abbreviation: SD = standard deviation.

### 3.2 Analysis of the Pulmonary Nodules

A total of 249 pulmonary nodules were analyzed, of which 186 (74.7%) were non‐metastasized and 63 (25.3%) were metastasized. Ten of the metastatic nodules (15.9%, 10/63) and six of the non‐metastatic nodules (3.2%, 6/186) were proven by pathology. Within the nodules without histological examination, the incidence of CT‐diagnosed metastasis was 22.7% [(63–10)/(249–16)]. Table 2 shows the characteristics and associated imaging findings of the nodules. A large proportion of the nodules were smaller than 6 mm (86.3%) with the metastatic nodules being larger than the non‐metastatic ones (*p* < 0.001). There were more perifissural nodules in the metastatic group than in the non‐metastatic group (23.8% vs. 15.1%, *p* = 0.11), while within the perifissural nodules, 34.9% (15/43) revealed metastasis. Additionally, more metastatic nodules were related to the presence of pleural effusion and lymphadenopathy (*p* < 0.001). Over half of the nodules (58.2%, 145/249) were round, and 73.1% (106/145) of them were benign.

**TABLE 2:** Characteristics of the nodules and associated imaging findings (*N*, %).

	Non‐metastatic nodules (*N* = 186, 74.7%)	Metastatic nodules (*N* = 63, 25.3%)	Total (*N* = 249)	*p*
**Size**				< 0.001
< 6 mm	171 (91.9)	44 (69.8)	215 (86.3)	
≥ 6 mm	15 (8.1)	19 (30.2)	34 (13.7)	
**Location (lobe)**				0.184
Right upper lobe	38 (20.4)	12 (19.0)	50 (20.1)	
Right middle lobe	43 (23.1)	9 (14.3)	52 20.9)	
Right lower lobe	44 (23.7)	20 (31.7)	64 (25.7)	
Left upper lobe	13 (7.6)	7 (11.3)	20 (8.0)	
Left lingual lobe	8 (4.3)	6 (9.5)	14 (5.6)	
Left lower lobe	40 (21.5)	9 (14.3)	49 (19.7)	
**Location (related to carina)**				0.464
Above carina	39 (21.0)	16 (25.4)	55 (22.1)	
Below carina	147 (79.0)	47 (74.6)	194 (77.9)	
**Shape**				0.849
Round	106 (57.0)	36 (61.9)	145 (58.2)	
Oval/lentiform	39 (21.0)	13 (20.6)	52 (20.9)	
Triangular	25 (13.4)	6 (9.5)	31 (12.4)	
Polygonal	16 (8.6)	5 (7.9)	21 (8.4)	
**Margin**				0.711
Smooth	184 (99.5)	63 (100)	248 (99.6)	
Non‐smooth	1 (0.5)	0	1 (0.4)	
**Attachment**				0.171
None	65 (34.9)	22 (34.9)	87 (34.9)	
Pleura	32 (17.2)	8 (12.7)	40 (16.1)	
Fissure	34 (18.3)	20 (31.7)	54 (21.7)	
Vessels	55 (29.6)	13 (20.6)	68 (27.3)	
**Pleural tag**				0.319
None	229 (92.0)	61 (96.9)	229 (92.0)	
Tag to pleura	18 (7.2)	1 (1.6)	18 (7.2)	
Tag to interlobular septum	1 (0.5)	1 (1.6)	2 (0.8)	
**Perifissural nodule (PFN)**				0.112
No	158 (84.9)	48 (76.2)	206 (82.7)	
Yes	28 (15.1)	15 (23.8)	43 (17.3)	
**Pleural effusion**				< 0.001
None	184 (98.9)	54 (85.7)	238 (95.6)	
Yes	2 (1.1)	9 (14.3)	11 (4.4)	
**Mediastinal/hilar lymphadenopathy**				< 0.001
None	185 (99.5)	52 (82.5)	237 (95.2)	
Yes	1 (0.5)	11 (17.5)	12 (4.8)	

Abbreviation: SD = standard deviation.

### 3.3 Predictors of Metastasis

We evaluated the nodular features significantly associated with metastases using univariate logistic regression (Table 3). Nodule ≥ 6 mm (OR = 4.9; 95% CI, 2.3–10.5; *p* < 0.001), pleural effusion (OR = 15.3; 95% CI, 3.2–73.1; *p* < 0.001) and lymphadenopathy (OR = 39.2; 95% CI, 4.9–309.9; *p* < 0.001) were associated with metastases.

**TABLE 3:** Univariate logistic regression analysis in discriminating pulmonary metastasis.

Variables	Odds ratio (95% confidence interval)	*p*
**Size (6 mm)**		
< 6 mm	Reference	
≥ 6 mm	4.92 (2.32–10.46)	< 0.001
**Location**		
Above carina	Reference	
Below carina	0.78 (0.40–1.52)	0.465
**Shape**		
Round	Reference	
Oval	0.91 (0.44–1.87)	0.790
Triangular	0.65 (0.25–1.71)	0.385
Polygonal	0.85 (0.29–2.47)	0.765
**Shape (round)**		
Round	Reference	
Others	0.82 (0.45–1.46)	0.495
**Attachment**		
None	Reference	
Pleura	0.74 (0.30–1.84)	0.516
Fissure	1.74 (0.83–3.62	1.140
Vessels	0.70 (0.32–6.51)	0.440
**Perifissural nodule (PFN)**		
No	Reference	
Yes	1.76 (0.87–3.57)	0.115
**Pleural effusion**		
None	Reference	
Yes (ipsilateral)	15.33 (3.22–73.11)	0.001
**Mediatinal/hilar lymphadenopathy**		
None	Reference	
Yes	39.12 (4.94–309.93)	0.001

Multivariate analysis was performed with both nodular and clinical features (Table 4). Controlling for the other variables in the model, larger nodular size, mediastinal/hilar lymphadenopathy, higher clinical stage, and triple‐negative breast cancer (TNBC) were all independently correlated with pulmonary metastases. Nodule ≥ 6 mm (OR = 5.0; 95% CI, 2.1–12.0; *p* < 0.001), and lymphadenopathy (OR = 12.9; 95% CI, 1.4–118.4; *p* = 0.02) were predictive of metastasis. Nodules (≥ 6 mm) were assessed as weak evidence to rule in metastasis, and the results were as follows: **+L**R, 3.74: accuracy, 76.3%; sensitivity, 30.2%; specificity, 91.9%; PPV, 55.9%; and NPV, 79.5%. Considering the clinical stages, Stages III (OR = 6.0; 95% CI, 1.6–22.8; *p* = 0.01) and IV (OR = 7.1; 95% CI, 2.3–22.1; *p* < 0.001) had a higher risk than stage I disease. TNBC had an odds ratio of 3.5 compared to pure HR‐positive cancer for pulmonary metastasis (95% CI, 1.0–11.8; *p* = 0.0047). Other radiological findings including location, shape, margin, and pleural tag were not associated with metastasis.

**TABLE 4:** Multivariate logistic regression of lung metastasis analysis in discriminating pulmonary metastasis.

Variables	Odds ratio (95% confidence interval)	*p*
**Size (6 mm)**		
< 6 mm	Reference	
≥ 6 mm	4.92 (2.32–10.46)	< 0.001
**Clinical stage**		
1	Reference	
2	2.41 (0.80–7.28)	0.119
3	6.00 (1.58–22.82)	0.009
4	7.06 (2.26–22.05)	0.001
**Pleural effusion**		
No	Reference	
Yes	3.84 (0.63–23.48)	0.145
**Mediastinal lymphadenopathy**		
No	Reference	
Yes	12.88 (1.40–118.40)	0.024
**Tumor grades**		
1 + 2	Reference	
3	0.65 (0.32–1.32)	0.230
**Breast cancer subtype**		
Hormone receptor (+), HER2 (−)	Reference	
Hormone receptor (+), HER2 (+)	1.41 (0.61–3.29)	0.420
Hormone receptor (−), HER2 (+)	0.98 (0.35–2.75)	0.966
Triple‐negative	3.47 (1.02–11.82)	0.047

## 4 Discussion

In the absence of formal guidelines, it is challenging for radiologists to diagnose pulmonary metastases when they encounter only a few (*n* ≤ 5) small pulmonary nodules (< 10 mm) on staging CT in breast cancer patients. We conducted this study to assess the clinical and imaging features related to metastases for these subjects and found that larger nodules (≥ 6 mm), the presence of mediastinal/hilar lymphadenopathy, a higher clinical stage, and the triple‐negative breast cancer subtype were independent risk factors of metastases.

In our study cohort, the distributions of clinical stage and breast cancer subtype were slightly different from the distributions obtained from large databases of Taiwan and the United States [11, 12] because fewer early‐stage breast cancer patients at our hospital underwent preoperative CT evaluation. Less Stage I and II diseases in our patient cohort (29.9% and 36.6%) was noted than the usual distribution in our country (37.0% and 43.4%) [4] and more HER2‐positive tumors were present. This was consistent with the National Comprehensive Cancer Network (NCCN) guidelines that additional imaging studies (such as CT) other than mammography and ultrasound are considered only in the presence of signs and symptoms of metastatic disease in early‐stage breast cancer and preoperative systemic therapy [3]. Breast cancer subtypes are known to be associated with different metastatic patterns, with HER2‐positive and TNBC more likely to metastasize to the lung [13, 14]. In the present study, 40.0%(6/15) of TNBC and 27.1%(19/70) of HER2‐positive tumors showed pulmonary metastases; this data is consistent with a previous study showing 31% and 25%, respectively [15]. In our multivariate regression model, only TNBC showed a significant risk of pulmonary metastases. HER2‐positive did not show a significant risk, which may be due to subject selection bias. The available evidence on risks for distant metastasis comes mainly from patients with early‐stage breast cancer who have received concurrent treatment with surgery, chemotherapy, and hormonal therapy [16]. Studies on the risks of pulmonary metastases at first diagnosis of breast cancer are limited.

Typical radiological findings of pulmonary metastases are multiple well‐defined, spherical lesions in a peripheral distribution and lower‐lobe predominance [5, 17, 18]; however, in our study, nodular shape, margin, and location did not show a significant connection with metastases. In general, smaller pulmonary nodules, of which simple pulmonary nodules accounted for a large population, provide limited morphological features. The larger the nodule, the more obvious its morphological features. Careful examination of morphological features could provide more information in the diagnosis of the etiology of pulmonary nodules [19]. PFNs are typically considered benign in lung cancer screening [8]. However, FPNs significance and optimal management in oncology patients remain unclear. In our study with all the patients having breast cancer, 23.8% of metastatic nodules were classified as typical PFN and the metastatic rate of PFNs was 34.9% (15/43), although PFN was not a predictor of metastasis (*p* = 0.11). Gloia Pernicka et al. [20] evaluated 62 oncology patients with a total of 112 PFNs and found that PFNs were stable or smaller in 59 patients (95.2%) after a median follow‐up of 5.7 years. They concluded that stable (23/112)/smaller (86/112) PFNs can be considered benign. However, since almost half (30/62) of the study cohort was receiving chemotherapy during the study period, smaller PFNs may also represent metastases that respond to chemotherapy. The best method for classifying nodules remains in comparison with previous study images to assess growth and morphological changes of the nodule [19]. Interestingly, PFNs occasionally exhibit transient rapid growth [21]. If doubt arises about the etiology of a nodule, short‐term follow‐up may be a good strategy, consistent with previous recommendations for nodule management.

The size of pulmonary nodules is consistently a significant indicator of malignancy in both screening CT and diagnostic CT for patients with extrapulmonary malignancy [6, 7, 22, 23, 24, 25]. This finding was also present in our study. In our study, 55.9% (19/34) of the nodules (≥ 6 mm) showed pulmonary metastases, and nodules (≥ 6 mm) were assessed as weak evidence to rule in metastasis. Additionally, it is worthwhile noting that the metastatic rate of the nodules less than 6 mm in our study was 20.8% (44/215), and in a prior study, the malignancy rate of small pulmonary nodules (≤ 4 mm) in oncologic patients was 28% [26]; hence, follow‐up of these small nodules is required. According to the study performed by Kerri‐Ann et al. [27], the doubling time of pulmonary metastasis from breast cancer was 82–199 days. Tumor growth is typically expressed in terms of volume doubling time, defined as the time taken from the nodule to double in volume or to increase 26% in diameter [28]. Therefore, follow‐up CT for small nodules (< 6 mm) in 6 months and nodules (≥ 6 to < 8 mm) in 3 months would be appropriate. For nodules (≧ 8 mm), diagnostic CT or PET/CT or tissue sampling may be considered, which is consistent with recommendations for growing nodule management [9, 29].

There are several limitations in our study. The study subjects were selected because not all the breast cancer patients had received at least two chest CT studies. Besides, as retrospective, observational research, the protocol of image acquisition, follow‐up intervals, and treatment regimens were not consistent. Another limitation is that the determination of metastases was mainly based on radiological evaluation of size change rather than pathological examination. Determination of metastases based on a change in the size of a nodule is empirical. Additionally, observational bias cannot be avoided with visual analysis and manual measurement. Without pathological confirmation, the metastatic potential of these nodules could be overestimated or underestimated. Finally, in the multivariate logistic regression of our study, some variables seem to overlap with each other. Pleural effusion and mediastinal lymphadenopathy may contribute to the clinical stage. It would be ideal to design a prospective study with consistent study protocols using pathological examination to confirm the nature of the nodules. Further guidelines for nodular management based on risk stratification are needed.

## Conflicts of Interest

The authors declare no conflicts of interest.

## Data Availability

The data that support the findings of this study are available on request from the corresponding author. The data are not publicly available due to privacy or ethical restrictions.
